# Maternal plasma viral load and neutralizing/enhancing antibodies in vertical transmission of HIV: A non-randomized prospective study

**DOI:** 10.1186/1743-422X-2-15

**Published:** 2005-02-24

**Authors:** Paul Kamara, Loyda Melendez-Guerrero, Miguel Arroyo, Heidi Weiss, Pauline Jolly

**Affiliations:** 1 Department of Epidemiology, School of Public Health, University of Alabama at Birmingham, 1665 University Blvd., Ryals Building, Room 217, Birmingham AL 35294-0022, USA; 2 Department of Microbiology and Medical Zoology, University of Puerto Rico, Medical Sciences Campus, School of Medicine, San Juan, Puerto Rico; 3 US Military HIV Research Program, Walter Reed Army Institute of Research, Division of Retrovirology, Silver Spring, MD 20910, USA; 4 Breast Center, Baylor College of Medicine, One Baylor Plaza, MS: BCM 600, 335A, Houston, TX 77030, USA

**Keywords:** HIV vertical transmission, HIV neutralization, maternal viral load, HIV enhancement

## Abstract

**Background:**

We examined the association and interaction between maternal viral load and antibodies in vertical transmission of HIV in a non-randomized prospective study of 43 HIV-1 infected pregnant women who attended the San Juan City Hospital, Puerto Rico, and their 45 newborn infants. The women and infants received antiretroviral therapy.

**Methods:**

A nested PCR assay of the HIV-1 envelope V3 region and infant PBMC culture were performed to determine HIV status of the infants. Maternal and infant plasma were tested for HIV neutralization or enhancement in monocyte-derived macrophages.

**Results:**

Twelve (26.7%) infants were positive by the HIV V3 PCR assay and 3 of the 12 were also positive by culture. There was a trend of agreement between high maternal viral load and HIV transmission by multivariate analysis (OR = 2.5, CI = 0.92, p = 0.0681). Both maternal and infant plasma significantly (p = 0.001 for both) reduced HIV replication at 10^-1 ^dilution compared with HIV negative plasma. Infant plasma neutralized HIV (p = 0.001) at 10^-2 ^dilution but maternal plasma lost neutralizing effect at this dilution. At 10^-3 ^dilution both maternal and infant plasma increased virus replication above that obtained with HIV negative plasma but only the increase by maternal plasma was statistically significant (p = 0.005). There were good agreements in enhancing activity in plasma between mother-infant pairs, but there was no significant association between HIV enhancement by maternal plasma and vertical transmission.

**Conclusion:**

Although not statistically significant, the trend of association between maternal viral load and maternal-infant transmission of HIV supports the finding that viral load is a predictor of maternal-infant transmission. Both maternal and infant plasma neutralized HIV at low dilution and enhanced virus replication at high dilution. The antiretroviral treatments that the women received and the small sample size may have contributed to the lack of association between HIV enhancement by maternal plasma and vertical transmission.

## Background

The rate of HIV-1 infection has been increasing rapidly among women of childbearing age. At the end of 2003 women accounted for 50% of adults living with HIV/AIDS worldwide [1]. Consequently, the number of pediatric AIDS cases due primarily to perinatal (peripartum or intrapartum) transmission is rapidly increasing. Mother-to-child transmission accounts for more than 90% of all HIV infections in infants and children worldwide. In 2003 an estimated 2.1 million children under 15 years were living with HIV/AIDS [1]. Zidovudine (ZDV) given as either an intensive or short course regimen significantly reduces perinatal transmission [2,3]. However, because of its cost, ZDV is not always available in poorer countries of the world. Successful use of nevirapine therapy in preventing perinatal transmission offers hope for more affordable treatment for poor women worldwide [4,5]. However, in 2003, only one in ten pregnant women was offered services for preventing mother-to-child HIV transmission [1]. Further, whether treated with ZDV or nevirapine, a portion of HIV-positive women still transmit virus to their offspring vertically and the problem of maternal-infant transmission through breast milk remains unsolved. Therefore, there is need for continued studies of viral and immunological factors associated with maternal-infant transmission of HIV so that other effective and affordable strategies to prevent transmission may be developed.

Although some studies show no association between the presence of HIV neutralizing antibodies in maternal sera and the risk of perinatal transmission [6,7], other studies report a reduction in the risk of vertical transmission in pregnant women whose sera contain neutralizing antibodies to HIV [8]. A number of studies have indicated lower transmission rates from infected pregnant women with high antibody titer or with high affinity/avidity antibody to conserved portion of HIV-1 glycoprotein 41 [9], to the CD4 binding site [10] or the V3 loop of glycoprotein 120 [11,12], and to the p24 Gag protein [13]. Other studies have reported that non-transmitting mothers more frequently have such antibodies to their own virus than do transmitting mothers and that transmitting mothers rarely have neutralizing antibody against their own children's isolates [14,15]. In contrast, a study by St. Louis, et al. [16] found no evidence that anti-V3 loop antibody protected against perinatal transmission. Further, a study by Lallemant, et al. [17] showed that mothers with higher antibody titers to peptides corresponding to the V3 region of gp120 and the immunodominant domain of gp41 had a higher risk of perinatal transmission. The authors hypothesized that women who display the broadest antibody response to V3 may be experiencing the greatest viral turnover [18] which could make them more at risk for transmitting virus to their offspring.

In contrast to neutralizing antibodies, non-neutralizing antibodies may enhance HIV infection by binding to the virus and facilitating its uptake by cell types that carry immunoglobulin (Fc) or complement receptors. Antibodies that enhance HIV replication *in vitro *by either Fc gamma receptor- or complement receptor-mediated endocytosis (FcγR-ADE or C'-ADE) have been identified in sera from HIV-1-infected individuals [19-25] and from many gp120-vaccinated volunteers [19,20]. An *in vitro *study of antibody dependent enhancement (ADE) of HIV-1 infection in human term syncytiotrophoblast cell cultures suggested that both FcγR-ADE and C'-ADE may contribute to maternal-infant transmission of HIV-1 [26]. Pancino, et al. [27], reported that mother-infant transmission of HIV was associated with maternal antibodies to the envelope gp160 and to a highly conserved domain of the trans-membrane glycoprotein. Mann, et al. [28], observed that certain combinations of antibody subclasses occurred more frequently in mothers who transmitted HIV-1 to their offspring than in non-transmitters and suggested that ADE may occur in mother-infant transmission of HIV-1. However, C'-ADE was not found to be associated with maternal-infant transmission of HIV [29] and the role of FcγR-ADE in maternal-infant transmission has not been determined.

Maternal plasma virus load has been shown to be strongly associated with perinatal transmission of HIV [27,30,31] and it was reported that there is no absolute threshold of maternal viral load below which HIV transmission does not occur [32]. Although transmission did not occur at a threshold below 2,000 copies/ml [18,33] or below 1000 copies/ml [34], more recent meta-analysis has demonstrated that occasional transmission does occur below a viral load threshold of 1,000 copies/ml [35]. Another study indicated that viral load correlated with vertical transmission in women at the clinical stage A1 (asymptomatic) of infection [36].

However, the association of both viral load and enhancing activity (presumably by FcγR-ADE) in maternal plasma and vertical transmission of HIV has not formerly been examined. Thus, we examined the association of these factors (independently and combined) in vertical transmission using samples from mother-infant pairs from San Juan, Puerto Rico, previously described by Melendez-Guerrero et al [37]. Neutralization/enhancement of a R5 tropic strain of HIV-1 subtype B by maternal plasma was examined in monocyte-derived macrophage cultures.

## Results and Discussion

### Study sample

A cohort of 43 HIV-1 subtype B infected pregnant women attending antenatal clinic at the San Juan City Hospital, Puerto Rico, was enrolled into a prospective study from their first antenatal visit until delivery [37]. Eleven women were enrolled during their first trimester, 24 during their second trimester, and 8 during their third trimester of pregnancy. Approximately forty-five HIV-1 infected women gave birth at the San Juan City hospital during 1998. All of the women recruited into the study received some form of antiretroviral therapy as detailed and referenced [3,38-44] in Table 1. All of the infants were enrolled into the study shortly after delivery and most (86%) were also enrolled in the antiviral protocols during their first six weeks of life (Table 1). One mother had triplets, therefore, a total of 43 mothers and 45 infants (regarded as 45 mother-infant pairs) were enrolled.

**Table 1:** Number and percent of HIV positive pregnant women and infants assigned to the different AIDS Clinical Trails Group (ACTG) protocols or to zidovudine (ZDV)

**ACTG Protocol #**	**Number of women (%)**	**Dose and frequency**
185 [38]	16 (37.2)	**Mother**: ZDV according to 076 protocol [3] plus anti-HIV immune serum globulin (HIVIg) or immune globulin (Ig) (200 mg/kg) every 28 days followed by 1.0 mg/kg/hr continuous infusion during labor and delivery. **Infant**: HIVIg (200 mg/kg) or normal Ig within 12 hrs of birth plus oral ZDV syrup (2.0 mg/kg) every 6 hours after birth (beginning within 8–12 h) and continuing for 6 weeks.
249 [39]	4 (9.3)	**Mother**: Didanosine (ddi) IV (1.6 mg/kg) on day one, during pregnancy, followed by an oral dose (200 mg) one week after the initial dose. Oral ddi is then administered every 12 hrs until labor starts and every 12 hrs after delivery until 6 weeks post-partum. During labor and delivery patients receive a loading dose followed by continuous infusion. **Infant**: Oral ZDV syrup (2.0 mg/kg) every 6 hours after birth and continuing to week 6.
250 [40]	5 (11.6)	**Mother**: ZDV plus Nevirapine (200 mg/kg) single dose during labor. **Infant**: Oral ZDV syrup (2.0 mg/kg) every 6 hours after birth and continuing to week 6 plus single dose of Nevirapine (2 mg/kg) after birth.
296 [41]	3 (7.0)	**Mother: **ZDV as in protocol 185. **Infant**: Oral ZDV syrup (2.0 mg/kg) every 6 hours after birth and continuing to week 6.
316 [42]	9 (20.9)	**Mother: **Nevirapine (200 mg oral dose) or the corresponding placebo during delivery plus ZDV (as in 076). **Infant: **ZDV perinatal prophylaxis (2.0 mg/kg) plus single 2.0 mg/kg oral dose of Nevirapine or Nevirapine placebo administered between 48 and 72 hrs of life.
324 [43]	3 (7.0)	**Mother**: ZDV before and after delivery as is usual but oral administration of ZDV (300 mg) every 3 hrs, 3 doses total during delivery. **Infant**: Oral ZDV syrup (2.0 mg/kg) every 6 hours after birth and continuing to week 6.
332 [44]	1 (2.3)	**Mother: **Stravudine (d4T, 30–40 mg) during pregnancy and 0.05 mg/kg/hr during delivery in combination with 3TC 150 mg followed by 150 mg during delivery. **Infant: **Stravudine (d4T, 1 mg/kg) single dose on day 35–42, in combination with 3TC (2.0 mg/kg/dose).
ZDV [3]	2 (4.7)	**Mother: **ZDV (2.0 mg/kg) every 28 days followed by 1.0 mg/kg/hr continuous infusion during labor. **Infant**: Oral ZDV syrup (2.0 mg/kg) every 6 hours after birth and continuing to week 6.

### Demographic and clinical characteristics of the mothers and vertical transmission

The mean age of the mothers was 24 years (range 14–38 years; Table 2). Majority of the women (88.4%) were infected through heterosexual contact. Based on the 1993 CDC revised classification system for HIV infection and disease progression, only 6 of the mothers (those in the A3 and B3 categories) were classified as AIDS cases [45]. Approximately two-thirds (67.4%) of the mothers had viral load levels below 10,000 RNA copies/ml and were classified as having low viral loads (LVL) based on the categorization by Contopoulos-Ioannidis & Ioannidis [46]. The remaining 32.6% of mothers had viral loads above 10,000 RNA copies/ml and were classified as having high viral loads (HVL). CD4+ T cell counts in the women ranged from 23 to 1165 cells/mm^3 ^of blood with a mean count of 425 cells/mm^3^. Twenty-six of the 43 women delivered their babies by normal vaginal delivery; the remaining women had cesarean sections (Table 2).

**Table 2:** Age, source of infection, clinical status and transmission outcome for HIV-positive mothers

Variable	Number (%)
Age of mothers (years)^a^	
< 25	14 (32.6)
25–30	12 (27.9)
> 30	16 (37.2)
Missing	1 (2.3)
Source of infection	
Heterosexual contact	38 (88.4)
IV drug use	3 (7.0)
Unknown	2 (4.6)
Clinical status (CDC classification 45)	
A1	11 (25.6)
A2	14 (32.6)
A3	1 (2.3)
B1	2 (4.6)
B2	10 (23.3)
B3	5 (11.6)
Viral load (copies/ml)^b^	
<10,000 (range 83–9,078)	29 (67.4)
≥ 10,000 (range 10,220–484,703)	14 (32.6)
Mode of delivery	
Vaginal	26 (60.5)
Cesarean	17 (39.5)
Transmission outcome	
V3 PCR+ Infants	12 (26.7)
V3 PCR- Infants	33 (73.3)
Culture+ Infants	3^c ^(6.7)
Culture- Infants	42 (93.3)

^a^Mean age 24 years (range 14–28 years)

^b^Mean viral load 28,112 copies/ml (range = 83 – 484,703 copies/ml)

^c^HIV infection confirmed by PCR and culture

Twelve (26.7%) of 45 infants were HIV V3 positive indicating that HIV transmission had occurred (Table 2). However, only three of these twelve infants were also HIV culture positive. This infection rate of approximately 7.0% observed is similar to the rate of 8.3% observed for ZDV treated mothers and infants in the 076 study [4]. Based on the V3 PCR results an equal number of mothers with low and high viral load (6 in each group) transmitted HIV to their infants. The mean log viral load of transmitting mothers 3.77 + 0.31 (median = 3.77) was higher than of the non-transmitting mothers 3.54 + 0.15 (median = 3.44) but the difference was not statistically significant (p = 0.474). This is probably due to the small sample size. The mothers of the three infants who were HIV culture positive all had high viral load levels (484,703, 11,642 and 10,220 RNA copies/ml) but the levels in two of the three were close to the 10,000 RNA copies/ml cut-off value used to distinguish LVL from HVL. Since perinatal transmission occurs mostly at or during delivery, the viral load in the genital tract (which may be similar to maternal plasma level) [47] may be an important determinant in maternal-infant transmission.

The mothers of HIV V3 positive infants had a non-significantly higher mean CD4+ T count (526 cells/mm^3^, median = 508 cells/mm^3^) compared to non-transmitting mothers (mean CD4+ T count of 413 cells/mm^3^; median = 336 cells/mm^3^). Maternal CD4 cell counts have been shown to be a less effective predictor of transmission of HIV than viral load [18]. Further, most of the women in the study (86%) had CD4+T cell counts above the 200 cells/mm^3 ^level which is used to define AIDS. Only one of the three women whose infants were HIV culture positive had AIDS (CD4 = 149 cells/mm^3^). The other two women had CD4 counts of 510 and 538 cells/mm^3^.

### Neutralization/enhancement of HIV treated with maternal and infant plasma

The effect of plasma (at 10^-1 ^to 10^-3 ^dilutions) from the mothers (transmitters and non-transmitters) and infants (HIV V3+ and HIV V3-) on HIV replication was examined in the neutralization/enhancement assay (Table 3). HIV p24 antigen in culture fluids was determined using the coulter p24 antigen assay kit (Coulter, Miami FL) according to the manufacturer's instructions. The mean value of HIV p24 antigen by dilution was calculated from seronegative samples. The percent change (increase or decrease) in p24 antigen of the maternal or infant plasma from the mean of the seronegative samples was calculated. Neutralization was defined as 70% or greater reduction in HIV p24 antigen in cultures treated with HIV positive plasma compared with cultures treated with HIV negative plasma. Enhancement was defined as 100% or greater increase in p24 antigen in cultures treated with HIV positive plasma compared to the p24 level in cultures treated with HIV negative plasma. Traditionally enhancement of HIV in PBMC cultures has been defined as a 1.5 to 2.5-fold or greater increase in virus replication as a result of treatment with immune sera [21,25,48]. Table 3 shows that at 10^-1 ^dilution, over 90% of plasma samples from both groups of mothers (transmitters/non-transmitters) and infants (HIV V3+/ HIV V3-) neutralized HIV. At 10^-2 ^dilution the percentages of plasma that neutralized HIV dropped to approximately 75% for both groups of mothers and to 58% and 70% for HIV V3+ and HIV V3- infants respectively. The percentages again dropped at 10^-3 ^dilution to ≤ 67% for mothers and ≤ 33% for infants (Table 3). Chi-square or Fisher's exact test were used to compare the proportion of plasma from transmitter versus non-transmitter mothers and between HIV V3+ and HIV V3- infants that neutralized, enhanced or resulted in no change in HIV replication. No significant differences were found.

**Table 3:** Neutralization/enhancement of HIV infection by plasma from 12 transmitter and 33 non-transmitter mothers and 12 HIV V3 positive and 33 HIV V3 negative infants

Plasma Dilution and effect	Transmitter mothers Number (%)	Non-transmitter mothers Number (%)	HIV V3+ infants Number (%)	HIV V3- infants Number (%)
10-1				
Neutralized	11 (92)	31 (94)	11 (92)	30 (91)
Enhanced	0 (0)	0 (0)	0 (0)	0 (0)
No change	1 (8)	2 (6)	1 (8)	3 (9)
				
10-2				
Neutralized	9 (75)	25 (76)	7 (58)	23 (70)
Enhanced	1 (8)	2 (6)	2 (17)	1 (3)
No change	2 (17)	6 (18)	3 (25)	9 (27)
				
10-3				
Neutralized	8 (67)	19 (58)	4 (33)	9 (27)
Enhanced	0 (0)	5 (15)	4 (33)	7 (21)
No change	4 (33)	9 (27)	4 (33)	17 (52)

**Figure 1:**
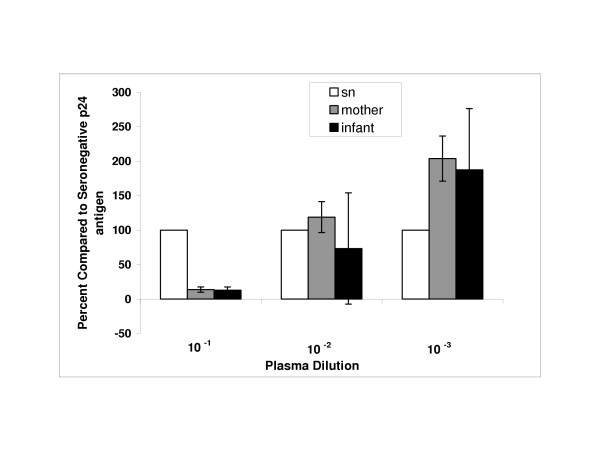
Neutralization/enhancement of HIV-1BaL by maternal and infant plasma diluted 10^-1 ^– 10^-3^. Virus replication (determined by p24 antigen (pg/ml) in culture fluids collected 2–4 days post-infection) is compared to replication of virus treated with plasma from HIV-1 seronegative (sn) women. The data represent the average of all maternal and infant samples. Two independent infections were conducted with each sample in duplicate (4 replicates) for each of 43 maternal and 45 infant plasma. Maternal and infant plasma significantly reduced HIV replication (p = 0.001 for both) at 10^-1 ^dilution compared to HIV negative sera. Infant plasma also significantly reduced HIV replication (p = 0.001) at 10^-2 ^dilution. At 10^-3 ^dilution maternal plasma significantly increased HIV replication (p = 0.005) above HIV negative sera and infant plasma showed a non-significant increase (88%) in HIV replication.

The mean percent change in p24 antigen and standard errors for mothers and infants were plotted for each group by plasma dilution (Figure 1). The Wilcoxon signed-ranked test was used to determine whether the percent change was significantly different from zero. Both the maternal and infant plasma significantly (p = 0.001 for both groups) reduced HIV replication at low (10^-1^) dilution when compared with HIV negative sera (Figure 1). At 10^-2 ^dilution the infant plasma still significantly (p = 0.001) reduced virus replication, but the maternal plasma lost neutralizing activity (Figure 1). At 10^-3 ^dilution, the maternal plasma significantly increased virus replication (p = 0.005) above seronegative plasma and the infant plasma showed a non-significant increase (88%) in HIV replication (Figure 1). These findings of neutralization by plasma of HIV positive individuals at low dilutions and enhancement at higher dilutions are similar to data published by Jolly and Weiss [49], which showed that neutralizing and enhancing antibodies can occur simultaneously in sera of HIV-infected individuals. If neutralizing antibody is present, enhancement is seen only at high dilutions, whereas, if only enhancing antibody is present, enhancement is observed without, or at low, plasma/serum dilutions.

### Enhancing activity in plasma from mothers and infants

Sixty-nine percent (31/45) of plasma from mother-infant pairs were also tested in the neutralization/enhancement assay at higher dilutions (10^-4 ^to 10^-6^). Examination of enhancing activity in plasma of mothers and infants showed that there were good agreements (60% or greater) in enhancement status between mother-infant pairs. For example, at 10^-4 ^dilution, the plasma of 7 infants of 9 mothers (78%) whose plasma enhanced HIV replication also exhibited enhancement, and at 10^-6 ^dilution, the plasma of 8 infants of 10 mothers (80%) whose plasma enhanced HIV replication also exhibited enhancement (data not shown). Comparison of p24 antigen between transmitter and non-transmitter mothers or their infants at 10^-4 ^to 10^-6 ^dilutions showed no significant difference between the two groups of mothers or infants. The lack of association of enhancing activity and HIV transmission in this study is similar to the findings for C'-ADE by Gras, et al. [29]. We examined complement-independent (presumably FcγR-mediated antibody dependent) enhancement in primary human macrophages because we thought that this type of enhancement would be more relevant to HIV clinical disease and transmission. FcγR-mediated enhancement is characteristic of diseases such as dengue and feline infectious peritonitis for which ADE has been best demonstrated to occur [50,51]. In addition, macrophages are important target cells for infection and replication *in vivo *by most HIV-1 variants [52-54]. However, our results showed no association between enhancing activity in maternal or infant plasma and maternal-infant transmission of HIV. The negative results could in part be due to the various anti-retroviral protocols to which the women were assigned.

### Correlation between maternal and infant p24 antigen levels and maternal viral load with maternal and infant p24 antigen

Using Spearman's correlation, we examined the association between maternal viral load and enhancing activity in vertical transmission of HIV by using mothers' characteristics (viral load and p24 antigen value) and the infants' p24 antigen values. A correlation matrix showed a positive association between mothers' p24 antigen values and those of their infants (Table 4). However, low positive or no associations were found between maternal viral load and maternal p24 antigen values at low dilutions (10^-1 ^– 10^-3^) and low negative associations at higher dilutions (10^-4 ^– 10^-6^). There were low positive associations between maternal viral load and the p24 antigen values of their infants at low dilutions (10^-1 ^– 10^-3^) and low negative correlations at higher dilutions except at 10^-5 ^dilution (Table 4). However, none of the values was statistically significant.

**Table 4:** Correlation (Spearman) of maternal and infant p24 antigen levels and maternal viral load with maternal and infant p24 antigen

Plasma dilution	p24 antigen (M vs I) Correlation^a ^log (p-value)^b^	MVL and Mp24 Correlation^c ^log (p-value)^b^	MVL and Ip24 Correlation^d ^log (p-value)^b^
10^-1^	0.412 (0.005)	0.12 (0.448)	0.09 (0.562)
10^-2^	0.59 (0.0001)	0.18 (0.249)	0.11 (0.473)
10^-3^	0.72 (0.0001)	0.14 (0.355)	0.04 (0.832)
10^-4^	0.40 (0.028)	-0.15 (0.423)	-0.16 (0.394)
10^-5^	0.75 (0.0001)	-0.10 (0.581)	0.31 (0.091)
10^-6^	0.69 (0.0001)	-0.34 (0.064)	-0.31 (0.089)

M = mother; I = infant; MVL = maternal viral load; Mp24 = maternal p24 antigen; Ip24 = infant p24 antigen

^a^Spearman correlation coefficient between log values of maternal and infant p24 antigen.

^b^p-value to test for statistically significant correlation.

^c^Spearman correlation coefficient between log values of maternal viral load and maternal p24 antigen.

^d^Spearman correlation coefficient between log values of maternal viral load and infant p24 antigen.

### Maternal viral load and enhancing antibodies as predictors of vertical transmission of HIV

Univariate analysis of type of treatment, stage of HIV disease, method of delivery and CD4+ T cell count of the mothers indicated no significant association with vertical transmission of HIV (p > 0.05). The combined effect of maternal viral load and enhancing antibodies as potential risk factors for the vertical transmission of HIV-1 was examined in a multivariable model. Log viral load was treated as a continuous variable in the model and enhancement was categorized as enhancement versus neutralization. There was a trend of association between maternal viral load and transmission of HIV-1 so that the higher the viral load, the more likely mothers were to transmit HIV-1 to their infants (OR= 2.5, CI= 0.93 – 6.67, p = 0.0681). This is in agreement with other studies discussed earlier that show viral load as a predictor of maternal-infant transmission [27,30,31]. The non-significant result in this study is probably due to the small sample size. Also, twice as many mothers (29) in this study had low plasma HIV RNA levels (<10,000 RNA copies/ml) compared to those with high viral load levels (14 mothers). The preponderance of women with low viral load levels may be the result of the effect of the different antiretroviral protocols that the women were given during pregnancy. Whereas Dickover, et al. [30] showed that women given zidovudine during gestation showed an eight-fold median decrease in plasma HIV RNA levels (p < 0.001), Sperling, et al., [32] have shown that ZDV treatment had only a minimal effect in decreasing maternal HIV RNA levels. In this study all of the women except one (given stravudine) received ZDV treatment and in most cases ZDV was given along with one other anti-retroviral drug or with HIVIg (Table 1). There was no significant association between enhancing activity (based on p24 antigen values) in the plasma of mothers who transmitted HIV-1 to their infants based on the HIV V3+ status of the infants (OR = 0.92, CI = 0.41 – 2.07, p = 0.8492).

## Conclusion

In agreement with published data, multivariate analysis showed a trend of association between maternal viral load and maternal-infant transmission of HIV. The non-significant difference in the mean log viral load of transmitting and non-transmitting mothers is probably due to the small sample size. No significant associations were found between HIV antiretroviral treatment protocols, classification of HIV disease, method of infant delivery and CD4+ T cell count of the mothers and vertical transmission of HIV. Both maternal and infant plasma significantly neutralized HIV infection at low (10^-1^) dilution and enhanced virus replication at higher dilution (10^-3^). Neutralizing and enhancing antibodies can occur together in the blood of HIV positive individuals and the neutralizing effect can be lost at high plasma dilution.

There were good agreements in the neutralizing or enhancing activity of the plasma from mothers-infant pairs. That is, when plasma of the mothers neutralized or enhanced HIV infection, their infants' plasma showed similar activity. However, there was no significant association between virus enhancement by maternal plasma and vertical transmission of HIV. Thus, enhancing activity in plasma of these HIV-infected mothers was not a dominant factor in vertical transmission of HIV.

## Methods

### Collection, processing and testing of maternal and infant blood samples for HIV

Blood samples were taken from the mothers during each trimester of pregnancy and during delivery. Blood samples from the infants were collected from the umbilical cord at birth and at 1–2, 3–4 and 5–12 months. Blood was collected in vacutainer tubes containing ACD anticoagulant and centrifuged at 2500 rpm for 15 minutes. The plasma was stored frozen (-20°C) for use in maternal viral load and HIV neutralization/enhancement assays. The remaining blood was diluted in phosphate-buffered saline (PBS) pH 7.4 and processed by Ficoll-hypaque density gradient for cell isolation. For virus isolation, one million cells from each mother or infant were co-cultured with HIV seronegative donor cells previously stimulated by PHA as described in the ACTG virology manual [55]. The remaining cells (10^5^) were stored frozen at -85°C. The HIV status of the infants was determined using a nested PCR assay of the HIV-1 envelope variable (V3) region as previously described [37,56]. This V3 PCR assay was conducted in duplicates and repeated on the infant samples at 1–2 months, 3–4 months and 5–11 months. Culture for HIV was repeated on infant samples collected at 3, 6 and 12 months and all except 3 infants were culture negative.

### Determination of maternal viral load

HIV RNA copies in maternal plasma was determined by the amplicor HIV monitor test (Roche Diagnostics, Branchwater, NJ, USA) at the Puerto Rico ACTG-certified laboratory [37]. A 142 base-pair sequence in the HIV *gag *gene was amplified by RT-PCR for viral load determination. The mean log viral load was calculated and compared between transmitting and non-transmitting mothers to determine the effect of maternal viral load on HIV vertical transmission.

### Preparation and titration of HIV-1BaL for the neutralization/enhancement assay

HIV-1BaL stock was prepared in primary macrophages as reported previously by Jolly [57]. Briefly, HIV-1BaL supernatant fluid (1 × 10^4.6 ^TCID_50_/ml) obtained from the NIH AIDS Research and Reference Reagent Program was used to inoculate fresh cultures of macrophages grown on 75 ml tissue culture flasks. The cultures were washed 24 hours later and incubated with fresh media. Supernatant fluids were harvested at 7 and 14 days post-infection, clarified by centrifugation at 1800 rpm for 10 minutes and used as stock virus for these studies. The stock contained 5 × 10^5 ^TCID_50_/ml.

### Neutralization/enhancement assay

A neutralization/enhancement assay was conducted using maternal or infant samples and HIV-1BaL. Maternal plasma samples collected during the third trimester of pregnancy were used in these assays since most prenatal HIV infections occur in the third trimester [58]. Briefly, 10-fold dilutions of heat inactivated (56°C for 30 min) plasma samples were mixed 1:1 with 10^3 ^TCID_50_/ml of virus and pre-incubated at 37°C for 30 min. The mixtures were then inoculated into replicate cultures of monocyte-derived macrophages as prepared previously [57] in 8 well chamber slides and incubated at 37°C for 6 hours in a 5% CO_2 _incubator. The inocula were removed and the cells washed, and incubated with fresh media for up to 8 days. Culture supernatant fluids were then collected on days 2, 4, 6, and 8 and tested for p24 antigen using the Coulter assay (Coulter, Miami, FL, USA). Cultures treated with HIV-negative sera were used as controls. All maternal and infant plasma samples were tested at three 10-fold dilutions; 69% of samples were tested up through six 10-fold dilutions. Two independent assays were conducted for each maternal or infant plasma sample and each sample was run in duplicate on each assay (4 replicates).

### Statistical analysis

Descriptive statistics such as mean, median, range were calculated to summarize maternal characteristics such as viral load, CD4+ T cell count and p24 antigen levels. The number of HIV vertical transmission among infants was summarized and the univariate association of maternal characteristics, such as, treatment (type of antiretroviral therapy), stage of disease based on the 1993 CDC classification [45], method of delivery (C-section vs. vaginal), and CD4+ T cell count, with HIV transmission in infants was evaluated using the Fisher's exact test. The simultaneous effect of maternal viral load and p24 levels on infant transmission was evaluated using the logistic regression model. Odds ratio and 95% confidence intervals were calculated for the effect of both factors in the model.

Correlation between maternal versus infant p24 antigen levels and between maternal viral load and maternal or infant p24 antigen levels was evaluated using the Spearman's correlation coefficient. Enhancement, decrease (neutralization), or no change in p24 levels compared to seronegative control was assessed for mother-infant pairs. The distribution of virus p24 antigen was compared between transmitting vs. non-transmitting mothers and between HIV V3+ infants and HIV V3- infants using Fisher's exact test.

## Competing interests

The author(s) declare that they have no competing interests.

## Authors' contributions

LMG and PEJ were involved in conception and design of the study, collection of the samples, interpretation of the data and drafting and critical review of the manuscript. PK and MA were involved in performing the laboratory experiments that resulted in the acquisition of the data, in data entry, and in drafting and revising the manuscript with LMG and PEJ. HLW was responsible for data analysis and interpretation along with PEJ, LMG and PK. All authors have read and approved the final manuscript.
